# The Reliability of Transcranial Magnetic Stimulation-Derived Corticomotor Inhibition as a Brain Health Evaluation Tool in Soccer Players

**DOI:** 10.1186/s40798-021-00399-3

**Published:** 2022-01-14

**Authors:** Thomas G. Di Virgilio, Magdalena Ietswaart, Ragul Selvamoorthy, Angus M. Hunter

**Affiliations:** 1 Physiology, Exercise and Nutrition Research Group, Faculty of Health Sciences and Sport, University of Stirling, Stirling, FK9 4LA Scotland, UK; 2 Psychology, Faculty of Natural Sciences, University of Stirling, Stirling, Scotland, UK

**Keywords:** TMS, Reliability, Soccer, Subconcussion, Concussion, Diagnostics

## Abstract

**Background:**

The suitability of corticomotor inhibition and corticospinal excitability to measure brain health outcomes and recovery of sport-related head impact (concussion and subconcussion) depends on good inter-day reliability, which is evaluated in this study. Transcranial magnetic stimulation (TMS) reliability in soccer players is assessed by comparing soccer players, for whom reliability on this measure may be reduced due to exposure to head impacts, to generally active individuals not engaged in contact sport.

**Methods:**

TMS-derived corticomotor inhibition and corticospinal excitability were recorded from the rectus femoris muscle during two testing sessions, spaced 1–2 weeks apart in 19 soccer players (SOC—age 22 ± 3 years) and 20 generally active (CON—age 24 ± 4 years) healthy volunteers. Inter-day reliability between the two time points was quantified by using intra-class correlation coefficients (ICC). Intra-group reliability and group differences on actual measurement values were also explored.

**Results:**

Good inter-day reliability was evident for corticomotor inhibition (ICC_SOC_ = 0.61; ICC_CON_ = 0.70) and corticospinal excitability (ICC_SOC_ = 0.59; ICC_CON_ = 0.70) in both generally active individuals and soccer players routinely exposed to sport-related head impacts. Corticomotor inhibition showed lower coefficients of variation than excitability for both groups (Inhib_SOC_ = 15.2%; Inhib_CON_ = 9.7%; Excitab_SOC_ = 41.6%; Excitab_CON_ = 39.5%). No group differences between soccer players and generally active individuals were found on the corticomotor inhibition value (*p* > 0.05), but levels of corticospinal excitability were significantly lower in soccer players (45.1 ± 20.8 vs 85.4 ± 6.2%Mmax, *p* < 0.0001). Corticomotor inhibition also showed excellent inter-rater reliability (ICC = 0.87).

**Conclusions:**

Corticomotor inhibition and corticospinal excitability are stable and maintain good degrees of reliability when assessed over different days in soccer players, despite their routine exposure to head impacts. However, based on intra-group reliability and group differences of the levels of excitability, we conclude that corticomotor inhibition is best suited for the evaluation of neuromuscular alterations associated with head impacts in contact sports.

## Keypoints

- The sequelae of repetitive impacts to the head in soccer and other contact sports are increasingly becoming a cause for concern.
- Non-invasive assessments of corticospinal excitatory and inhibitory mechanisms can help shed light on potential brain changes associated with subconcussive head impacts. However, no data exist on the reliability of such parameters in a contact sport context.
- The current study shows that parameters of corticospinal excitation and inhibition are stable and show good short-term test–retest reliability in soccer players. Inhibitory responses may be the better measure for assessments in a subconcussive context due to methodological limitations associated with corticospinal excitability.

## Introduction

Injuries of a concussive (and more recently subconcussive) nature have been at the center of attention for researchers, clinicians and sporting bodies alike. Such injuries have been shown to be associated with both acute and chronic impairments to cognitive function [1–3], motor control [4–6] and brain structure [1, 7]. However, commonly used methods to assess brain and motor function in a sporting context are either indirect (e.g. balance as a proxy for motor control) [8], or can be unreliable (e.g. cognitive function) [9]; in the latter case individuals, particularly athletes, are able to circumvent the tests in order to continue playing despite receiving a concussive injury [9]. With growing awareness of dangers surrounding repetitive concussive and subconcussive injuries in contact sport (including soccer), there has been a drive from researchers to provide direct and objective indications of brain function following such impacts.

Single pulse transcranial magnetic stimulation (TMS) allows researchers to quantify intracortical, corticospinal excitatory and inhibitory mechanisms governing motor skills [10]. Measures of excitation and inhibition have been shown to be altered by traumatic brain injuries (TBIs); more specifically, cortical silent period (cSP) appears increased, whilst corticospinal excitability is suppressed [11–13]. Our research group has shown that corticomotor inhibition is acutely and transiently modulated following a bout of soccer ball heading, suggesting increased GABA-ergic activity [14]; however, we are unaware of any studies examining TMS reliability in this particular population or in any other contact sport.

Various factors may affect repeatability of TMS-based parameters, including the positioning of the coil over the scalp, the target muscle being investigated, number of stimuli applied over the primary motor cortex (M1) and the stimulator intensity [10]. As such, it is unsurprising that studies assessing day-to-day reliability of corticomotor inhibition and corticospinal excitability show fair-to-excellent reliability (ICC values ranging from 0.52 to 0.92) [15–17]. Critically in a sporting context, it is possible that the repeatability of TMS-derived mechanisms may be particularly affected in those sports where players are routinely exposed to repetitive head impacts, as we have shown inhibitory mechanisms to change following just a single bout of subconcussive impacts (i.e. soccer heading and sparring in boxing) [14, 18].

Whether the test–retest reliability of TMS parameters depends on the population assessed is ambiguous. For example, whilst there is evidence suggesting that inhibitory and excitatory measures are reliable across the lifespan [16], other studies show poor reliability in a generally healthy elderly population [19]. To the best of our knowledge there is no evidence on the TMS reliability of comparable populations (e.g. different sports, male and females etc.); in particular, whether such test–retest reliability is good in those individuals who are routinely exposed to sport-related head impacts (in this case soccer players) is not yet known. Therefore, as cumulative exposure to subconcussive head impacts could possibly impact upon these measures, it is important to compare test–retest reliability in soccer players and generally active individuals. Furthermore, because routine impacts to the head could give rise to increased variation in TMS-dependent parameters, test–retest assessments should be undertaken with no heading exposure in between testing sessions.

The aim of this study was to explore the inter-day reliability of excitatory and inhibitory mechanisms in soccer players, to establish whether these measures are suitable in detecting potential subconcussive-dependent changes to the brain-to-muscle pathways. Based on previous findings [15–17], we can expect both parameters of motor control to exhibit fair-to-excellent test–retest reliability, but it is uncertain whether this is also the case for individuals routinely exposed to sport-related head impacts.

## Methodology

### Approvals and Recruitment

A total of 39 participants were recruited for the study via advertisements on university noticeboards and social media. Sample size was determined using a web app developed by Arifin, 2018 [20] and available at https://wnarifin.github.io/ssc/ssicc.html. Using a minimum acceptable reliability ICC of 0.6 (standard for “good” reliability), an alpha level of 0.05, 80% power and two repetitions per participant the recommended sample size is 18. Individuals participating in soccer were allocated in a group referred to as “SOC” (*N* = 19; 14 males, 5 females; age 22 ± 3 years; mass 72.9 ± 8.3 kg; height 175.4 ± 10.2 cm), and the generally active control participants in “CON” (*N* = 20; 16 males, 4 females; age 24.3 ± 4 years; mass 76.4 ± 13.6 kg; height 174.9 ± 9.8 cm). Participants in SOC ranged in terms of skills and abilities from recreational to playing for a club/University). At the time of recruitment soccer players headed the ball at least 15 times per week, whilst the control group were generally active individuals who did not partake in any contact sport. All participants were screened prior to taking part; potential candidates were excluded if they presented with any of the following: (1) history of brain injury resulting in loss of consciousness; (2) history of a neurological condition; (3) history of concussion in the 12 months prior to taking part; (4) family history of epilepsy; (5) current use of psychoactive recreational or prescription drugs; (6) do not normally head a soccer ball at least 15 times per week (SOC group only, self reported). The local Research Ethics Committee approved the study and procedures conformed to the guidelines set out by the Declaration of Helsinki. Written informed consent was obtained from all participants prior to taking part.

### Study Design

Participants were asked to refrain from vigorous physical activity, consuming alcohol, and caffeine or smoking for 24 h prior to each study session. Participants were also required to present to the laboratory fasted and were provided with a standardized breakfast. Prior to commencing data collection, participants reported to the laboratory for a familiarization session, during which they completed all outcome measures to acquaint them with the assessment procedures and minimize later learning effects. Following the practice session, participants reported to the laboratory for two further experimental days, spaced a minimum of one and a maximum of 2 weeks apart (mean follow up 11 ± 5 days).

### Maximal Torque Production, Electromyography and Femoral Nerve Stimulation

Maximal isometric voluntary contractions (MVC) were assessed from the quadriceps femoris of participants’ dominant leg (self-reported) at the beginning of each testing day. Participants first performed a series of warm up contractions (3 ×  ~ 50% (perceived) MVC and 3 ×  ~ 70% (perceived) MVC. Subsequently, participants were instructed to achieve peak torque and maintain the contraction for 5 s; all participants performed 3 contractions, with 60 s rest in between and consistent verbal encouragement was provided to ensure maximal effort. The highest torque output achieved was designated as MVC and stored [21].

MVCs alongside all other electromyography (EMG) and TMS measures (see below) were recorded with participants sitting with their dominant leg secured to a calibrated load cell of an isokinetic dynamometer (Biodex System 4, New York, NY, United States). Knee angle was set at 60° (0° being fully extended limb) and the arm of the dynamometer was set such that the axis of rotation was aligned with participants’ lateral femoral condyle.

Electromyographic activity was recorded from the rectus femoris using a wireless system (Biopac Systems, Inc. Goleta, CA, USA). Data were sampled at 2 kHz, and filtered using 500 Hz low and 1.0 Hz high band filters. Signals were analyzed offline (Acqknowledge, v3.9.1.6, Biopac Systems, Inc. Goleta, CA, USA). EMG activity was assessed using Ag/AgCl surface electrodes (Vermed, Devon, UK) with an intra-electrode distance of 2 cm positioned over rectus femoris; prior to electrode placement, the area of interest was shaved and abraded as per Surface Electromyography for the Non-Invasive Assessment of Muscles (SENIAM) guidelines [22]. The position of each electrode was marked with permanent ink to ensure consistent placement during subsequent visits.

Peripheral stimulation of the femoral motor nerve was administered using an electrical stimulator (Biopac Systems, Inc.). The stimulation site was identified by locating the femoral artery and placing a self-adhesive surface electrode (cathode) lateral to it, high over the femoral triangle, with the anode on the gluteus maximus. Single stimuli were delivered to the muscle while participants maintained a 20% MVC isometric contraction, and the intensity of stimulation was increased until a plateau in twitch amplitude and rectus femoris M-wave (Mmax) occurred. Supramaximal stimulation was delivered by increasing the final stimulator output intensity by a further 30%.

### Transcranial Magnetic Stimulation

Motor evoked potentials (MEPs) were elicited in the rectus femoris of the dominant leg via single pulse TMS and assessed using electromyographic (EMG) recordings. Single magnetic stimuli of 1 ms duration where applied over the contralateral primary motor cortex using a magnetic stimulator (Magstim 200^2^ unit, The Magstim Company Ltd., Whitland, UK) and a 110 mm double cone coil. Optimal coil location for generating MEPs was determined by placing the coil over the motor cortex, laterally to the vertex; the area where the largest MEP peak-to-peak amplitudes occurred was identified and marked on the scalp with ink [23]. The active motor threshold (aMT) for the quadriceps femoris was determined by increasing stimulator output from 10% by 5% increments, while the participant held a ~ 20% MVC until discernible MEPs were visible in at least half of the stimulations delivered over M1 [24]. Once this individual level was established, subsequent stimulations were delivered at 130% of aMT.

To assess corticomotor inhibition participants were required to perform MVCs of 5 s duration while a single TMS stimulation was delivered over the motor cortex. This was repeated three times with 60 s rest between contractions, as is common practice for MVC assessments [21]. Corticomotor inhibition was quantified as the silent period duration (cSP), taken from the stimulation artefact to the resumption of discernible, uninterrupted EMG activity from the muscle [14, 18]. Silent period duration was assessed by two independent raters and data were randomised (both group and time-points) to avoid any potential bias. Pre-stimulus EMG was measured 100 ms before each stimulation was delivered to ensure consistent muscle activation across testing days. Average raw EMG signals were root mean square (RMS) processed with average RMS calculated for a 100 ms window, as previously described [25]. Processed EMG values were then normalized to the maximal signal taken from the first MVC participants performed during testing sessions [21]. To further ensure consistency between testing days, MEPs generated by stimulating M1 during MVCs were also recorded and expressed as a percentage of the maximal response elicited by motor nerve stimulation (Mmax) [26].

During the assessment of corticospinal excitability, participants maintained a 20% MVC isometric contraction while 20 single TMS pulses, separated by 6 s, were delivered over the motor cortex. Corticospinal excitability was determined as the average MEP amplitude normalized to the Mmax [26]. We chose to assess cortical excitability and inhibition in the lower limbs rather than in the upper limbs because of its functional relevance; in a sporting environment, changes in lower limb may be more valid as they relate directly to gait and sporting activities [27, 28].

### Statistical Analysis

Data collected from the 2 laboratory visits were organized based on the timepoint at which they were recorded (SOC timepoint 1, SOC timepoint 2; CON timepoint 1, CON B timepoint 2). Normality of the data was checked using the Shapiro–Wilk test, data were normally distributed. Reliability of the measures within each population was quantified using intra-class correlation coefficients (ICC) and coefficients of variation (CVs) computed by SPSS (v21; IBM Corporation). ICC values for inter-day reliability were calculated using a 2-way random effect model (single measures—2,1), whilst ICC values for inter-rater reliability (for cSP) were calculated using a 2-way random effect model (average measures, 2,k) with pooled data. All ICC values were interpreted as following: ≤ 0.39 = poor; 0.4–0.59 = fair; 0.6–0.74 = good; 0.75–1 = excellent, as outlined by Cicchetti [29]. The CV values were calculated using the formula: (*σ*/*µ*) * 100; where *σ* is the standard deviation, and *µ* is the mean of the sample. CV differences between excitatory and inhibory responses were explored on scistat.com (https://www.scistat.com/statisticaltests/comparison_of_coefficientsofvariation.php#) using tests from Forkman [30]. Analysis for statistical differences between groups was carried out using two-factor repeated measures ANOVAs [2 groups (independent factor) × 2 time points (repeated measures factor)]. If significant differences were observed, Tukey’s post hoc tests were used to further explore effects. Mean cSP differences between raters were assessed using an independent samples *t* test. Statistical significance was set at *p* ≤ 0.05; data are expressed as means (± standard deviation) unless otherwise stated.

## Results

### Inter-day Reliability

Corticomotor inhibition appeared stable across timepoints, as ICC analysis showed good test–retest inter-day reliability both in generally active individuals (ICC_CON_ = 0.70) and in soccer players (ICC_SOC_ = 0.61) (Table 1). Corticospinal excitability showed good and fair inter-day reliability in generally active individuals (ICC_CON_ = 0.70) and in soccer players (ICC_SOC_ = 0.59), respectively.

**Table 1:** Intraclass correlation coefficients and coefficients of variation for corticomotor inhibition and corticospinal excitability

	ICC_(2,1)_ (95%CI)	CV
*Corticomotor inhibition*		
Soccer players	0.61 (0.22–0.83)	15.2%
Generally active	0.70 (0.39–0.87)	9.7%
*Corticospinal excitability*		
Soccer players	0.59 (0.19–0.82)	41.6%
Generally active	0.70 (0.53–0.93)	39.5%
*Inter-rater reliability*		
Corticomotor inhibition	0.87 (0.79–0.91)	R1 = 13.7%; R2 = 14.6%

*ICC* intraclass correlation coefficient, *95%CI* 95% confidence intervals, *CV* coefficient of variation, *R1* rater 1, *R2* rater 2

### Intra-group Reliability

Intra-group analysis of corticomotor inhibition showed moderate CVs (CV_SOC_ = 15.2%; CV_con_ = 9.7%) (Table 1). Intra-group analysis of corticospinal excitability showed high coefficients of variance within each of the groups (CV_SOC_ = 41.6%; CV_CON_ = 39.5%) (Table 1). Comparing inter-day variability between the two measures, CV values for corticomotor inhibition were statistically lower than those for corticospinal excitability for both in the soccer group and in the generally active group (*p* < 0.001).

### Corticomotor Inhibition Inter-rater Reliability

Inter-rater analysis for silent period duration showed excellent reliability (ICC = 0.87) with similar CVs between raters (Rater 1 CV = 13.7%; Rater 2 CV = 14.6%) (*p* = 0.585) (Table 1). No differences were observed between cSP measured by each rater (*p* = 0.843).

### Parameter Group Differences

**Fig. 1:**
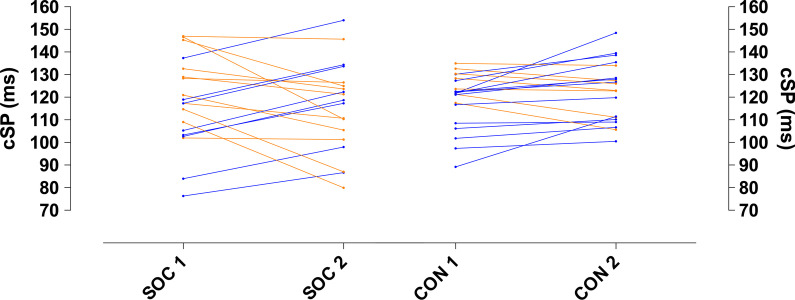
Corticomotor inhibition taken 100%MVC for each individual during each testing session. Blue dots represent participants who increased from time-point 1 to time-point 2, whilst orange dots participants who decreased between timepoints

**Fig. 2:**
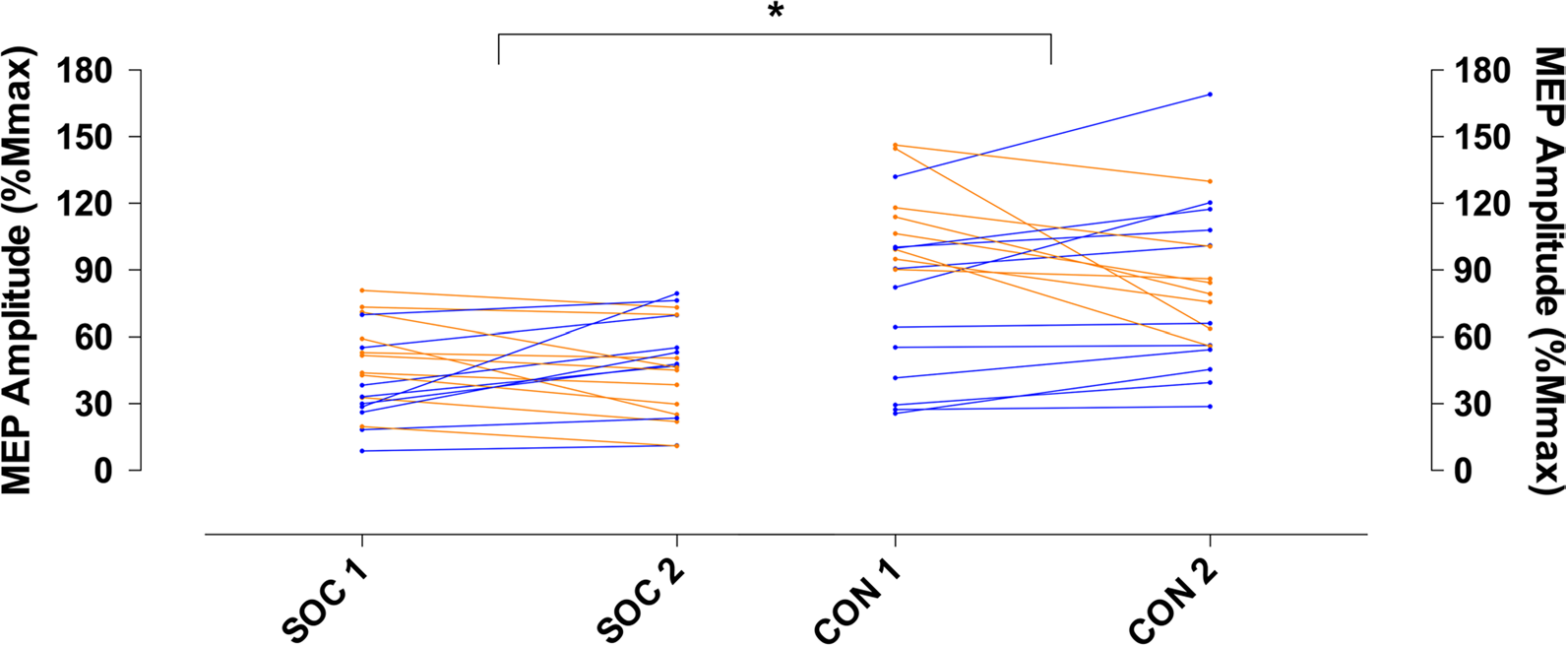
Corticospinal excitability taken at 20%MVC for each individual during each testing session. *denotes significant differences between groups (*p* < 0.05). Blue dots represent participants who increased from time-point 1 to time-point 2, whilst orange dots participants who decreased between timepoints

There were no differences between soccer players (116.8 ± 19.5 ms) and generally active individuals (120.7 ± 12.7 ms) when examining the actual cSP values, across both groups (*p* = 0.305) and time points (*p* = 0.795) (Fig. 1), nor were there any interaction effects (*p* = 0.443). In contrast, the excitatory parameter values showed differences between groups and timepoints (*p* < 0.0001; *F*_(3, 72)_ = 11.6; *η*^2^*p* = 0.32) (Fig. 2). Corticospinal excitability in the soccer group at timepoint 1 (SOC 1) was significantly lower than in the generally active group at time point 1 (CON 1, *p* = 0.0002; CI = 17.95–68.98), and at time point 2 (CON 2, *p* = 0.0007; CI = 13.68–64.72). Corticospinal excitability in the soccer group at time point 2 (SOC 2) was significantly lower than in the generally active group at time point 1 (CON 1, *p* = 0.0003; CI = 15.95–66.98), and at time point 2 (CON 2, *p* = 0.001; CI = 11.68–62.72).

### Silent Period Duration Pre-stimulus EMG, Motor Evoked Potentials and Maximal Torque Production

Pre-stimulus EMG showed no difference across both groups (*p* = 0.527) and time (*p* = 0.555), and no interaction effects (*p* = 0.443) (Table 2). Whilst motor evoked potentials showed no time and interaction effects (*p* = 0.376 and 0.865, respectively), the soccer group was significantly lower than the controls (*p* < 0.001; *F*_(1, 36)_ = 12.23; *η*^2^*p* = 0.145) (Table 2). Similarly, maximal torque production showed no time and interaction effects (*p* = 0.661 and 0.777, respectively), however soccer players were significantly lower than controls (*p* < 0.001; *F*_(1, 367)_ = 11.90; *η*^2^*p* = 0.139) (Table 2).

**Table 2:** Mean ± SD values for pre-stimulus EMG, MEP size and maximal torque production from the corticomotor inhibition assessments

	SOC		CON	
	T1	T2	T1	T2
Pre-stimulus EMG (%MVC)	48.56 ± 13.50	54.09 ± 15.73	50.61 ± 11.24	48.44 ± 8.78
MEP (%Mmax)	55.58 ± 26.95	63.90 ± 38.73	84.31 ± 31.49^a^	89.95 ± 38.01^a^
MVC (Nm)	164.79 ± 45.26	174.69 ± 56.56	213.25 ± 72.68^a^	216.17 ± 65.04^a^

*SOC* soccer group, *CON* control group, *T1* time point 1, *T2* time point 2

^a^Significant group difference

## Discussion

We have shown that corticomotor inhibition and corticospinal excitability show good inter-day reliability, not only in a general population as has been previously shown [16, 17, 31] but also in soccer players. These findings are significant as we show TMS-derived measures are stable despite soccer players’ routine exposure to head impacts through football heading. We suggest these inhibitory and excitatory parameters can be used to reliably assess health outcomes following concussion or subconcussion in soccer, and potentially in other contact sport athletes. The test re-test period chosen in this study is relevant in return to play scenarios where concussion recovery is monitored. TMS derived outcome measures are particularly relevant in such settings due to the objectivity and repeatability they offer.

Purely based on good inter-day reliability, our findings suggest both TMS derived measures offer good outcome measures in soccer players. However we show that intra-group variability in both populations was lower for corticospinal inhibition than for corticospinal excitability, indicating that excitability within each group was variable. Corticospinal excitability furthermore exhibited group differences: excitation in the soccer group was consistently lower than the same parameter in the generally active group. Therefore, the present study provides evidence that corticomotor inhibition is the better TMS-derived parameter for the evaluation of the consequences of concussive and subconcussive impacts in soccer, based on inter-group variability and actual values. High coefficient of variation values for excitability compared with lower values or inhibition have also been shown in previous literature [17, 32]. Combined with the seemingly sports specific modulation of actual excitability values, as shown in this study, our findings further question the reliability of this parameter in the context of recovery and monitoring. It is of interest that systematic reviews and meta-analyses also suggest inhibitory responses to be better suited in quantifying cortical changes in the context of concussion and subconcussion [13, 33, 34]. Exposure to head impacts appears to affect GABAergic mechanisms, nevertheless, there needs to be a standardized methodological approach implemented before TMS can be used in this context [33].

Although values of inter-day reliability were somewhat lower for both TMS parameters in soccer players, because the 95% CI values for each group overlap, we can safely infer that this lower value in soccer players is not of significance compared with the generally active group (following Lu and Shara [35]). Critically, good day-to-day reproducibility was found in the soccer player when individuals are assessed in a “healthy state” (i.e. not exposed to impacts to the head). This is of interest in light of previous findings where excitatory and inhibitory mechanisms were found to be affected by concussive and subconcussive impacts [12–14, 18, 36]. That said, in the absence of data on long term reliability of TMS-evoked parameters in sports where players are routinely exposed to concussive/subconcussive impacts (e.g. soccer, rugby, American football), studies are still needed to establish the chronic excitation/inhibition reproducibility in each of the different contact sport disciplines over a longer period of time than evaluated here in soccer players.

It is furthermore important to highlight the inherent limitations of TMS that preclude a definite standpoint on its usefulness in a concussive/subconcussive context [33]. One of the general issues with TMS studies is that there is no consensus on the specific methodology to be used for the recording of excitatory and inhibitory parameters, as can be noted by observing the wide range of procedures implemented in the published literature. Most studies use differing stimulator output intensities, and furthermore, the level of muscle activation is not kept constant: some use contractions well below submaximal intensities (10–30% MVC) [17, 31], others perform excitability testing whilst the muscle is at rest [37]. Lastly, the target muscle also differed from study to study, ranging from small muscles in the upper limbs (First dorsal interossei, Abductor pollicis brevis) [31, 38] to large muscles in the lower limbs (Vastus lateralis, Gastrocnemius) [17, 39]. A combination of such factors may affect measurement stability, resulting in a wide range of reliability values and problematic interpretations of electrophysiological changes following injury.

With regards to methodological limitations, it should be noted that in the current study the values shown for corticospinal excitability are normalized to the Mmax of the same muscle, whilst previous studies presented raw values [15, 16]. Corticospinal excitability, quantified as MEP amplitude, is usually expressed as a ratio of the maximal excitability of the muscle [40]. TMS is not able to activate the whole motor neuron pool in M1, therefore it is useful to know what proportion of the pool is being activated when the stimuli are delivered over the scalp [40]. However, measuring Mmax could introduce degrees of error, as slight changes of electrode placement during testing will affect the resulting waveform, ultimately reducing the repeatability of the excitatory parameter. It is possibly because of this methodological limitation that we show excitation for some participants to be above the maximal excitability of the muscle. Future studies may therefore want to consider using other parameters of excitation which do not rely on Mmax normalisation for interpretation (e.g. Input–Output curves) [34]. In contrast, in the current study (as was the case in our previous work [14, 18]; and work by Goodall et al. [26] corticomotor inhibition was assessed during maximal contraction of the leg. By measuring corticomotor inhibition at 100% MVC we ensure recruitment of a motor unit pool large enough to provide a full understanding of what is occurring within the corticospinal tract [40], even though this limits the number of feasible repetitions. Measuring cSP using a submaximal muscle contraction may not be as sensitive since a smaller pool of motor units is recruited, possibly reducing the impact of GABA inhibitory mechanisms on EMG signals. In turn, this would make cSP measurements less sensitive in detecting subtle and transient corticospinal changes. Furthermore, whilst participants in both groups showed comparable levels of muscle activity when tested for corticomotor inhibition, the control group was able to generate more torque than soccer players. Muscle contraction intensity is shown to affect MEP size, with greater intensities resulting in larger MEPs [41]. Higher torque production in the control group would partly explain the discrepancy in excitation between groups, although it is then unclear why pre-stimulus EMG activity is not significantly different between soccer players and the control group. Conversely, the silent period used to quantify corticomotor inhibition appears unaffected by contraction intensity as long as the stimulator output is kept consistent [42]. These findings are corroborated by our results as we show comparable inhibitory levels between groups regardless of torque production, further emphasizing the usefulness of this parameter.

Corticomotor inhibition in the current study was quantified manually by two independent raters. Manual assessment of silent period duration is thought to be less objective than automated methods which may affect inter-rater reliability [43]. Our findings show excellent agreement between raters, suggesting that adopting a standardised approach to quantify silent period duration can ensure high repeatability across researchers. Furthermore, it is likely that manual and automated methods are comparable to each other, as published evidence show good levels of agreement for the procedures [43, 44]. Ultimately, manual quantification of silent period is more time consuming; future studies should consider using automated methods, especially as freely available tools to do so are becoming more common [43]. Lastly, we acknowledge that the group-level findings provided in this study do not necessarily translate to reliability at an individual basis. Although TMS-based parameters show promise for use in concussion and subconcussion contexts, large scale studies are needed to understand whether the technique can be applied as a diagnostic tool at an individual case-by-case basis.

More recently subconcussion in general, and soccer heading in particular, have become of societal importance due to the potential links between these head impacts and decreased brain function and an increased chance of neurodegenerative disease [45]. As such, sport-related routine head impact is being studied more and more by researchers wanting to understand and explain how impacts such as ball heading or tackling may affect brain function. In particular, more researchers are concentrating on subconcussive-dependent changes to the neuromuscular system, as it appears perturbed by both concussive and subconcussive exposure [18, 33]. Therefore, the findings shown in this study are of use to future researchers when establishing what tools and methodology are best suited to study subconcussive head impacts.

## Conclusion

This is the first study to demonstrate that parameters of cortical inhibition and excitability show good short-term test–retest reliability in soccer players and the general population alike. Therefore, these TMS-derived parameters are suitable in quantifying motor function in sports involving routine exposure to head impact. However, TMS-assessed excitation appears to be suppressed in soccer players as found in this study. Combined with the methodological limitations of TMS-assessed excitation seemingly resulting in increased variability across individuals in general, we conclude that corticomotor inhibition is the better measure for assessment and monitoring in the context of concussive and subconcussive injuries.

## Data Availability

The datasets used and analysed during the current study are available from the corresponding author on reasonable request.

## Abbreviations

Ag/AgCl: Silver/silver chloride
aMT: Active motor threshold
ANOVA: Analysis of variance
CI: Confidence intervals
CON: Control group
cSP: Corticosilent period/corticomotor inhibition
CV: Coefficient of variation
EMG: Electromyography
GABA: Gamma-aminobutyric acid
ICC: Intra-class correlation coefficient
M1: Primary motor cortex
MEP: Motor evoked potential
Mmax: M-wave
MT: Motor threshold
MVC: Maximal voluntary contraction
RMS: Root mean square
SENIAM: Surface Electromyography for the Non-Invasive Assessment of Muscles
SOC: Soccer group
TBI: Traumatic brain injury
TMS: Transcranial magnetic stimulation
